# In Vivo Visualization of the Pericardium Meridian with Fluorescent Dyes

**DOI:** 10.1155/2021/5581227

**Published:** 2021-03-29

**Authors:** Tongju Li, Bruce Qing Tang, Wei-Bo Zhang, Minyi Zhao, Qingchuan Hu, Andrew Ahn

**Affiliations:** ^1^Ennova Institute of Life Science and Technology, ENN Group, Langfang, China; ^2^Institute of Acupuncture and Moxibustion, China Academy of Chinese Medical Sciences, Beijing, China; ^3^Division of General Medicine and Primary Care, Beth Israel Deaconess Medical Center, Harvard Medical School, Boston, MA, USA

## Abstract

The anatomical basis of acupuncture meridians continues to be enigmatic. Although much attention has been placed on potential correlations with inter/intramuscular fascia or lower electrical impedance, animal studies performed in the past 40 years have shown that tracer dyes—specifically Tc-99m pertechnetate—injected at strategic skin points generate linear migrations closely aligning with acupuncture meridians. To evaluate whether this phenomenon is also observable in humans, we injected two fluorescent dyes—fluorescein sodium and indocyanine green (ICG)—into the dermal layer both at acupuncture points (PC5, PC6, and PC7) and a nonacupoint control. Fifteen healthy volunteers were enrolled in this study. Of the 19 trials of fluorescein injected at PC6, 15 (79%) were associated with slow diffusion of the dye proximally along a path matching closely with the pericardium meridian. Furthermore, the dye emerged and coalesced proximally at exactly acupoint PC3. Injections of ICG at the acupoints PC5, PC6, or PC7 showed a similar trajectory close to the injection site but diverged when migrating proximally, failing converge on acupoint PC3. Injections of either dye at an adjacent PC6-control did not generate any notable linear pathway. Both ultrasound imaging and vein-locating device did not reveal any corresponding vessels (arterial or venous) at the visualized tracer pathway but did demonstrate correlations with intermuscular fascia.

## 1. Introduction

Meridian theory is a fundamental part of traditional Chinese medicine (TCM). To many TCM practitioners, acupuncture meridians are not mere conceptual constructs but physically authentic anatomical structures. Consequently, numerous attempts have been made since the 1950s to demonstrate the objective existence of meridians with the use of modern technologies. Many experiments resorted to searching for distinctive biophysical characteristics of the meridians and acupoints, including electrical, acoustic, thermal, optical, magnetic, isotopic, and myoelectric properties [1]. Others focused on anatomical correlates, including primo vessels (“Bonghan ducts”) [2], intermuscular fascia [2], and neurovascular bundles [3].

Despite these years of effort to date, there is no conclusive scientific evidence for the existence of acupuncture points or meridians [4]. The challenge in substantiating something as elusive as the acupuncture meridian arises from the fact that the physiological basis of acupuncture therapy itself remains unclear [1]. The search for acupuncture anatomy therefore becomes an endeavor to not only identify physical anatomical correlates but also gain physiological insights into how acupuncture may mechanistically operate [5]. Thus far, three prominent hypotheses have emerged as potential candidates.

The first hypothesis incorporates the loose connective tissue network [6]. The landmark Langevin et al.'s study in 2002 reported that nearly 80% of acupoints corresponded to intermuscular/intramuscular fascial planes [7]. Subsequently, studies investigated the physiological implications of this correlation and found evidence that biomechanical stress/tension induced by needle twirling in loose connective tissue may reduce inflammation via local purinergic (adenosine) signaling pathways [8, 9]. The second hypothesis revolves around the electrical characteristics of acupuncture points/meridians. As early as the 1950s, points and meridians were identified as localized sites with increased electrical conductivity, reduced resistance, and increased capacitance [1, 10, 11]. Furthermore, these electrically distinct points were associated with unique histological, functional properties such as increased gap junctions [12, 13], nNOS expression [12, 14], and subcutaneous collagenous bands [15]. The third hypothesis, which has garnered more interest recently, is the notion that acupoints are sites with increased pressure sensitization, which are further accentuated by the presence of clinical pathology [16–19]. These points are localized by extravasation of Evans blue dye into the skin interstitium, and needling these points generated significant improvements in the clinical condition compared with needling adjacent controls [16].

These three hypotheses have largely been pursued independently without much effort to consolidate or integrate the separate findings into a unifying process. Identifying a potential link between these mechanisms may provide an important holistic understanding of acupuncture's physiological effects. One potential candidate for this role is the flow of interstitial fluids through connective tissues [15]. Past studies found that injection of tracer dyes at acupuncture points led to linear migration of the dye along a path aligning with acupuncture meridians [11, 20–28]. The observed migratory speed, path trajectory, and selective association with technetium-99 (^99m^TcO_4_, and not other nuclear dyes) led authors to speculate that paths were attributed to a yet undescribed channel with low hydraulic resistance through the extracellular matrix [29, 30] and not to known vessels—vein or lymphatics [22, 28]. Moreover, these studies were replicated multiple times in numerous laboratories, although these were largely confined to animal subjects.

To re-evaluate this tracer-dye phenomenon, we employed clinical-grade, fluorescent contrast agents—fluorescein sodium [31] and indocyanine green (ICG) [32]—at the pericardium channel on the forearms of healthy human volunteers. This study hypothesizes that fluorescent dyes will migrate along meridian pathways when injected at acupuncture points but not at adjacent controls. Additional diagnostic imaging techniques, such as ultrasound and infrared vein detectors, were utilized to elaborate the anatomical structures potentially attributable to these migratory paths.

## 2. Materials and Methods

### 2.1. Participants

A total of 15 healthy volunteers (11 men, 4 women) were recruited for the study. The average age was 37.5 ± 7.5 years with a range of 30 to 56 years. Individuals who had any chronic medical condition as determined by a recent clinical medical examination were excluded. The study was approved by the Chinese Ethics Committee of Registering Clinical Trials (approval No. ChiECRCT20200040) and was conducted in accord with the Declaration of Helsinki. Written informed consent was obtained from all subjects before the study.

### 2.2. Injection of Fluorescent Dyes and Fluorescence Observation

Pericardium (PC) meridian and the acupoints were identified on the forearm from the wrist to the elbow by two TCM physicians (Drs Zhao and Hu) according to “Nomenclature and location of acupuncture points” (GB/T 12346-2006) issued by the Standardization Administration of China. Acupuncture point locations were reached by consensus and further refined or confirmed with the use of a low-electrical impedance locator device (Acupuncture Meridian Locator WQ6F30; Beijing, China) [14, 33]. The acupuncture points were demarcated with the use of two triangular stickers or circled with a marker pen aligned orthogonally to the meridian.

Both fluorescein sodium and ICG dyes were used. In 12 subjects, 0.1 mL of 20% fluorescein sodium (Wuzhou Pharmaceutical Co., Ltd., Guangxi, China; Product batch number: 181102) was injected intradermally at the left PC6 (Neiguan acupoint), and in a subset of 5 individuals, the right PC6 was also injected at a later date. Extra care was placed in ensuring that the injections were placed intradermally and not subcutaneously. When the fluorescence disappeared completely, some participates also received the 2^nd^ or the 3^rd^ injection at PC6. To assess a different fluorescent dye and other PC points, 0.1 mL of 5 mg/mL ICG (Weicai Pharmaceutical Co., Ltd, Liao Ning, China) was injected at PC5 (Jianshi), PC6, or PC7(Daling) in 6 subjects. An adjacent control for PC6 located midway between PC6 and LU7 (approximately 1 cm radial to PC6) was tested with fluorescein injection in 7 subjects.

Fluorescence of fluorescein sodium was observed on the forearm with a laser beam of wavelength 455 nm and illumination power of approximately 0.5 mW. Photographic images were obtained and recorded with a long-pass filter of 500 nm to filter out the illumination light. ICG dye was excited with 780 nm laser and recorded with a mobile phone with a long-pass filter greater than 780 nm or with a PDE imager (model C9830; Hamamatsu Photonics, Japan). This imager utilizes a LED light source (760 nm) and a CCD camera capturing photons with wavelengths greater than 820 nm [34]. Depending on the rate of migration of the fluorescent tracer, images were obtained at intervals sufficient to capture the dynamic progression/clearance of the dye and for as long as 10 hours (fluorescein) or more than 2 days (ICG). These images were obtained in a dark room with all other light sources turned off.

### 2.3. Vessels Visualization with a Vein Finder

To delineate the anatomic structure through which fluorescent dyes migrated, a vein illumination device (Projection Vein Finder V800P; Shenzhen Vivolight Medical Device & Technology Co., Ltd., China) was used over the same location where the dye pathway was observed. The fluorescein tracer and vein images were photographed separately and together for comparison. Because the vein finder utilizes infrared light (940 nm, compared to 450 nm wavelength for fluorescein) and operates through different visualization mechanisms (hemoglobin absorption and not fluorescence), there was no confounding of imaging between the different methods.

### 2.4. Ultrasound Imaging of Vessels and Fluorescent Traces

Ultrasound imaging was also used to demarcate the anatomical structures that co-located with the dye pathway. Because the fluorescent dye is invisible to ultrasound imaging, the linear dye trajectory was marked on the skin with a pen and overlaid with a thin metal wire that generated a narrow, linear shadow in the acquired B-mode images. Ultrasound scanning was performed both transversely and longitudinally along the metal wire with an ultrasound diagnosis system (Philips HD15 ultrasound system). Ultrasound gel pad (MIBO Technology Co. Ltd, Shenzhen, China) was used to provide better visualization of the superficial skin layers and improved delineation of the metal wire location. Color Doppler was also obtained to identify vascular structures.

### 2.5. Statistical Analysis

The data were statistically analyzed with SPSS Statistics for Windows (version 22.0; IBM Corp., Armonk, NY, USA). Ages of volunteers are presented as mean ± standard deviation. Fisher's exact test was applied to compare the ratios of injections producing fluorescent trajectories along meridian to total injections in different conditions. Bivariate correlation analysis with Pearson coefficients was applied to evaluate the association of age and the outcome. *P* values (2-sided) less than 0.05 were considered significant.

## 3. Results

### 3.1. Visualization with Fluorescein Sodium

Intradermal injection of fluorescein sodium at PC6 was performed in 12 study participants. In 15 of the 19 trials, a linear fluorescence migration was observed proceeding proximally, closely aligning with the pericardium meridian. All 15 tracer pathways were within 1 cm distance of the TCM-defined pericardium channel through the whole course of visualized trajectories.

**Figure 1:**
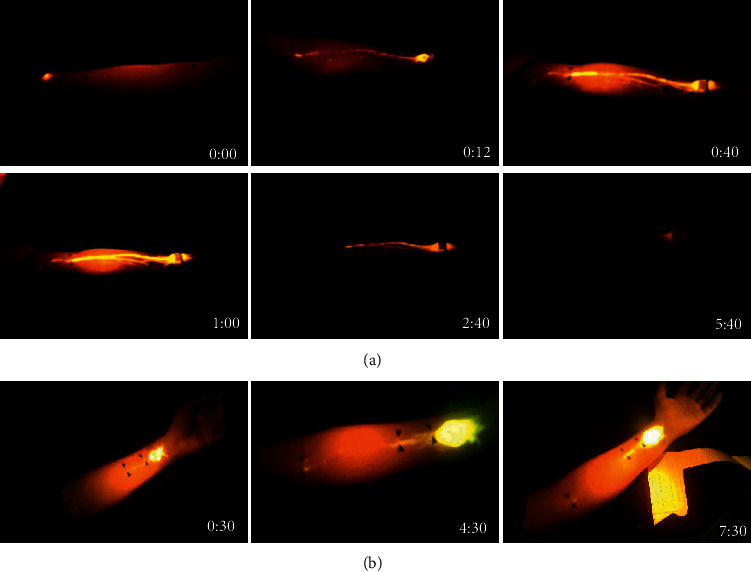
Development of fluorescent lines. Migration of fluorescein from PC6 with respect to time after injection (hour: minute) as shown in right lower corners. In series (a), migration was rapid, while in series (b), migration was slower. In (a), a clear line was observed as early as 10 minutes, peaked at 60 minutes, then diminished thereafter. Of note, in (b), fluorescence at PC3 preceded appearance of the linear path between PC3 and PC4.

**Figure 2:**
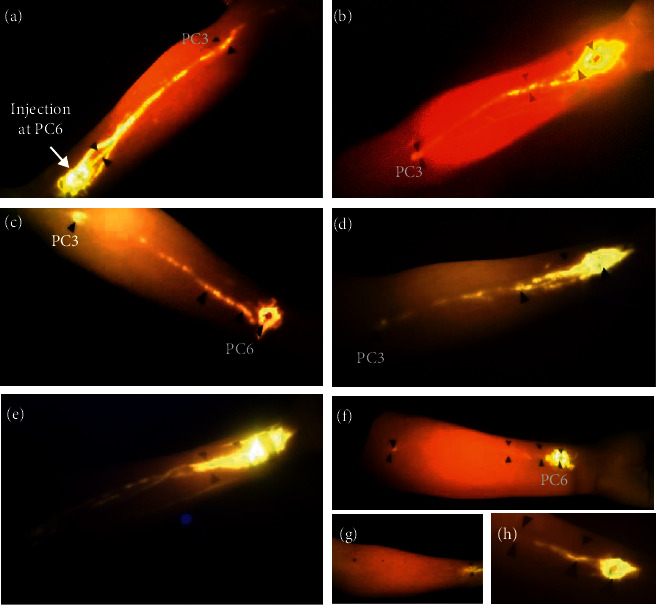
Variations in fluorescein migratory paths. Examples are provided to reveal variations in dye migrations. (a, b) Fluorescent signals were strong and quickly formed linear trajectories. Additional collateral lines were observed accompanying the main meridian-associated line. (c, d) Intermittent, broken lines formed along PC meridian within 30 minutes after injection. (e) Dye was also visualized in blood vessels. (f) Linear paths formed slowly 1 hour after injection and was weakly visible; nevertheless, PC3 appeared approximately 40 minutes before the linear path could be visualized. Similarly, in other cases, only PC3 fluoresced, while no linear path was observed (not included here). (g, h) No linear trajectories or point fluorescence at PC3 was seen.

From a temporal perspective, weak fluorescence points started to appear near PC6, and continuous or broken lines began to form 10 to 60 minutes after injection. Over time, they became increasingly distinct and passed through the demarcated acupoints PC5 and PC4, terminating at acupoint PC3 (Figures 1 and 2). A distinct pathway more proximal to PC3 was not observed with the fluorescein sodium dye. The fluorescent path in the forearm otherwise began to diffuse and fade away slowly over hours. The rate of tracer formation and disappearance varied from person to person. In some participants, the fluorescent line could be clearly seen approximately 10 minutes after injection with increased fluorescent intensities peaking at 1 hour; the line would last for up to 6 hours (Figures 1(a), 2(a) and 2(b)). In other volunteers, no continuous line could be seen, although PC3 alone was illuminated (*n* = 5). The fluorescence at PC3 was notably distinct: it fluoresced earlier than the region between PC4 and PC3, was markedly brighter with a circular appearance (larger diameter than the width of the linear path), and also lasted longer with persistent fluorescence lasting up to 8–10 hours (Figures 1(b) and 2(c)).

**Figure 3:**
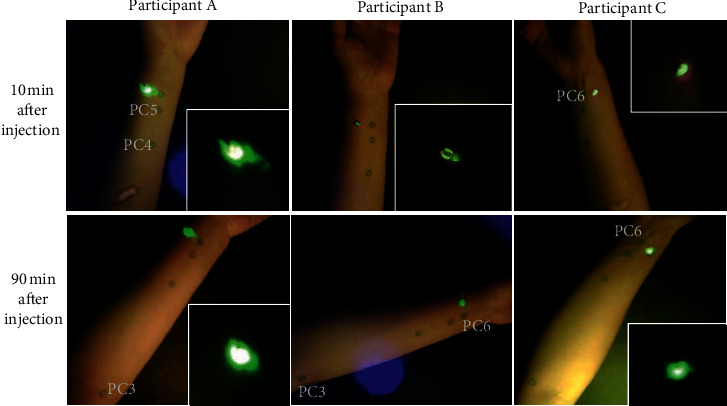
Injection of fluorescein in nonacupoint (PC6 control). Fluorescein injected at a nonmeridian point did not produce a linear pathway, even 90 minutes after injection.

In 7 trials where fluorescein sodium was injected into an adjacent nonacupoint control (nearly 1 cm radial to PC6), no evident presence of a linear trajectory was found proximally in the forearm even after 90 minutes of observation (Figure 3). To rule out a person-specific effect, in one of these volunteers, the PC6 on the contralateral arm was injected and a clear linear tracer pathway through PC5 and PC4 was observed, similar to that observed in the 15 out of 19 PC6 injection trials mentioned previously. This observed difference (PC6 vs. control point; linear path vs. no path) was statistically significant (*P* < 0.001, Fisher's exact test).

Diffusion of the fluorescent dye into blood vessels was also seen in some individuals during skin injection. Obvious trace along veins was found either alongside (Figure 2(b)) or without the presence of (Figure 2(f)) the linear dye pathway. The fluorescence from the blood vessels disappeared quicker and earlier than the dye migrating along the PC meridian. In addition, one hour after the injection, the urine became yellow and partially oily, indicating much of the fluorescein was being directly excreted through blood circulation and the urine. At the end of 4-5 hours, urine returned to baseline and no trace of fluorescein could be obviously seen.

### 3.2. Visualization with ICG

**Figure 4:**
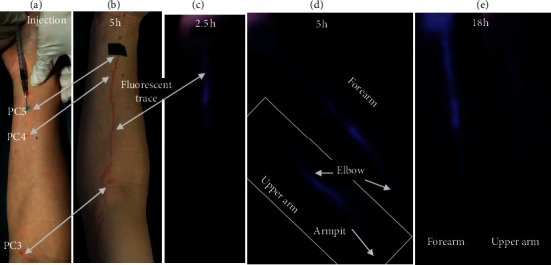
ICG migratory path after injection at PC5. After injection of ICG at PC5 (a), fluorescent line develops and is imaged here at 2.5, 5, and 18 hours (c–e). Five hours after injection, fluorescent lines are observed in both the forearm and upper arm (d). The fluorescent line was traced with a pen (b), passing by PC4 and PC3. Eighteen hours after injection, the fluorescence on the upper arm is still visible (e). The photo and video were taken with a mobile phone with long-pass filter above 780 nm.

**Figure 5:**
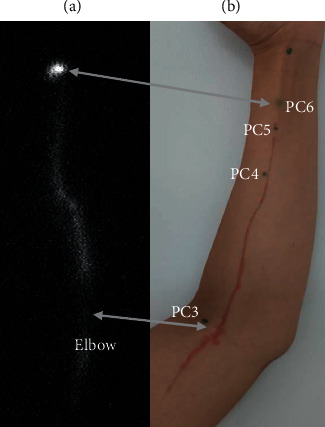
ICG fluorescent line 24 hours after injection at PC6 (a). The fluorescent line was traced and compared with acupoints PC5, PC4, and PC3, marked before injection (b) (black dots). These points and traced line closely approximated each other.

**Figure 6:**
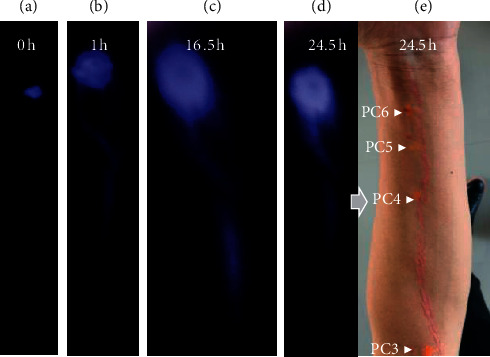
ICG fluorescent line after injection at PC7 (a). One hour after injection, the fluorescent line appears as shown in (b). Sixteen hours later, it is weaker (c). After 24.5 hours, the line in (d) was traced and compared with acupoints (e). PC5, PC4, and PC3, marked as pink cross, closely approximated to the red traced line.

ICG was injected at PC5 (Figure 4), PC6 (Figure 5), or PC7 (Figure 6)—each in a separate participant. Because excitation and emission wavelengths of ICG (∼800 nm) were in the near-infrared range, ICG fluorescence is able to be visualized at greater depths (up to 2 cm) compared with fluorescein sodium [32, 35]. Similar to fluorescein sodium, ICG produced fluorescence signals proceeding proximally along the PC meridian at an onset time of 6 minutes to 1 hour after injection. Unlike the fluorescein signal, ICG fluorescent paths were visible beyond the elbow (proximal to PC3) and were evidently progressing toward the axilla. Once the lines were fully established across the whole arm, they were traced with a pen under the excitation light. In most cases, PC3 and PC4 were within 1 cm distance from the traced line, suggesting possible correlation between the ICG pathway and the PC meridian. However, the ICG fluorescence was more diffuse compared with fluorescein, did not generate a highly distinct PC3, was diverted away from PC3 rather than passing directly through it (Figures 4 and 5), and generally persisted more than 48 hours.

### 3.3. Factors Associated with Dye Migration

Among the total 35 injections with fluorescein or ICG, 28 injections at acupoints produced 22 lines along PC, while 7 injections at the control point did not yield any linear projections. The breakdown of the results by injection site is shown in Table 1.

In total, 11 men and 4 women took part in the experiments. There were no statistical differences in the proportion of tracer migration paths associated with predetermined meridian/points between men and women (*P* = 0.208, Fisher's exact test). Age also did not correlate with the proportion of dye pathways associated with meridians (*P* = 0.814, bivariate correlation analysis).

### 3.4. Supplementary Imaging Techniques of PC6-Associated Pathway

**Figure 7:**
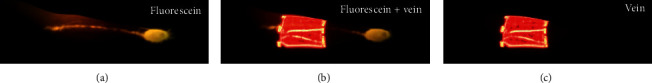
Fluorescent path covisualized with vein-imaging. (a) Fluorescence imaging only after fluorescein injection in PC6. (c) Vein Finder imaging only. (b) Overlay of both florescent line and vein-imaging revealing no clear overlap between the fluorescent pathway and visible vasculature.

After fluorescein path from acupoint PC6 was fully developed, a vein illumination device was used to assess whether the path aligned with superficial vasculatures. As seen in Figure 7, veins depicted as white on a background of pink did not overlap or coincide with the main florescence line shown in greenish yellow.

**Figure 8:**
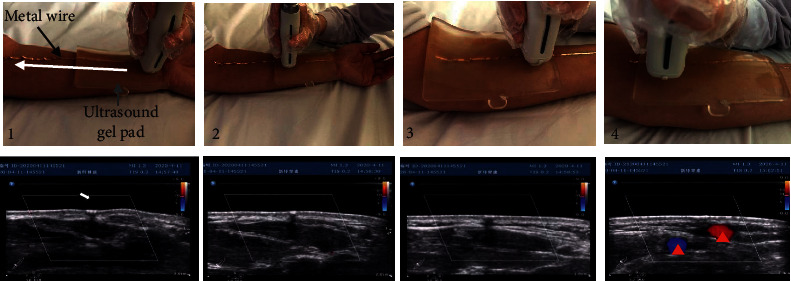
: Transverse ultrasound imaging of fluorescent line. As shown in upper images, metal wire was overlaid on the fluorescent line and fixed in place with a rectangular ultrasound gel pad. Transverse images were obtained as the ultrasound probe followed the course of the metallic wire from PC6 toward PC3. The metal wire is seen as an echogenic white dot (white arrow in left lower picture), casting a B-scan shadow. Color Doppler signal was only seen at the end of scanning (near elbow PC3) but not directly under the metallic wire.

Ultrasound images further corroborated the lack of association between any blood vessel and the fluorescent pathway. Because fluorescent dye is invisible in ultrasound imaging, a thin metal wire was placed over the previously observed fluorescent line and is visible as an echogenic dot casting a shadow down the B-scan (Figure 8). Ultrasound scanning was performed along the metal wire both longitudinally and transversely (Figure 8).

Figure 8 shows a representative result after transverse scanning of the forearm along the fluorescent line. For much of the path, the metallic wire was situated over the fascia located between flexor carpi radialis and flexor digitorum superficialis muscles (Figures.  8, 1–3). Color Doppler did not reveal any corresponding vasculature through most of the linear course from PC6 to PC3. It was only near PC3 that there were obvious Doppler signals to suggest presence of blood vessels in the proximity of the tracer pathway (red triangles in Figure 8).

## 4. Discussion

In this study, injection of fluorescein sodium into acupuncture points PC5, PC6, and PC7 was associated with slow diffusion of the dye proximally along a path aligning closely with the pericardium meridian. Furthermore, the dye emerged and ostensibly coalesced exactly where PC3 was demarcated beforehand. Injection of ICG at the acupoint PC6 showed a similar trajectory close to the injection site but diverged when migrating proximally and did not converge on acupoint PC3. Injections of either fluorescein or ICG at an adjacent PC6-control did not generate any notable linear pathway, at least in the timescale of our imaging acquisitions. Both ultrasound imaging and vein-locating device did not reveal any corresponding vessels (arterial or venous) at the visualized tracer pathways.

The study results reinforce the past studies that identified a slow-migrating pathway of tracer dyes along a linear path resembling acupuncture meridians [28]. Kovacs in 1992, for instance, hypodermically injected 4 different nuclear tracers—[^99m^Tc] sodium pertechnetate, [^201^Tl] thallium chloride, [^131^I] iodine sodium, and [^99m^Tc] rhenium sulfide—into both low-electrical resistance points and adjacent controls on the legs of adult dogs [22]. Only the [^99m^Tc] sodium pertechnetate injections into low-electrical resistance points gave rise to a linear radioactive pathway, which traveled at approximately 2.5 cm/minute over 11 cm distance, similar in scale as our study. Direct injections of the dyes into blood vessels led to a much more instantaneous appearance and subsequent disappearance of the trajectory in 10 seconds, while [^99m^Tc] rhenium sulfate—which has a predilection for lymphatics—was detected at axillary lymph nodes after 90 minutes in both acupoints and controls, without an evident linear pathway. For this reason, the authors concluded that veins or lymphatic vessels could not have been responsible for these trajectories [22]. Another study from China similarly found that fluorescein injected into acupuncture points have similarly reported visualization of interstitial channels along meridians in rats and mini pigs [36].

Our study is unique in incorporating fluorescent dyes in the study of an acupuncture meridian in healthy human volunteers. Compared with nuclear tracers, fluorescent dyes provide significant improvements in imaging resolution since the emitted photons during fluorescence imaging far exceed the amount released by gamma radiation in nuclear imaging at the doses considered safe for humans. Furthermore, fluorescent imaging does not require heavy machinery or the radioactive safety precautions needed for nuclear imaging and has thus enabled longer and more frequent monitoring of dye migration over time. Possibly for these reasons, this may be the first time that a dye was documented to aggregate at a proximal acupoint—specifically PC3—after injecting at a more distal site. To our knowledge, this represents the first evidence of a functional, dynamic link between meridian and point where both acupuncture structures were simultaneously visualized in a single test setting. The tight correspondences between the preimaging localization of PC3 (by acupuncturists) and the aggregation site of fluorescein were unanticipated and argued strongly for the specificity of fluorescein dye for localizing acupuncture-specific anatomy. Why this observation was seen with fluorescein and not with ICG remains unknown.

Despite its advantages, fluorescence imaging is not without limitations. The emitted photons from excited fluorescein at 500 nm range are prone to absorption and scattering by the skin and underlying tissues [32, 35]. Fluorescein dye was not well delineated at greater depths [31] and may not, for this reason, have been visualized proximal to the elbows or at the forearm in some individuals where it likely traversed at deeper levels. The fact that the dye re-emerged at PC3 in many suggests that the migratory pathways have a shared pattern across individuals of accessing superficial loci. The photons emitted from excited ICG, on the other hand, have wavelengths in the infrared (800 nm) range and are less apt to be attenuated by the skin and subcutaneous layer [35]. Consequently, ICG—when migrating beyond the injection site—was nearly universally seen at the forearm and upper arm. Although it co-localized closely with the pericardium meridian near the acupoint-injection site, it seemingly diverged away from the meridian as it migrated proximally and occasionally branched into several tributaries without ever manifesting at acupoint PC3 (in contrast to fluorescein).

The physicochemical characteristic of these dyes may account for these observed differences. ICG has historically been applied in certain clinical settings, specifically for imaging liver neoplasms [37], identifying thoracic duct [35], and detecting sentinel lymph nodes [38]. It is known to bind to albumin and enter the lymphatic vessels where it can accumulate at lymph nodes [32]. The ICG pathways observed in our experiment are thus likely to depict lymphatic vessels, and its lack of accumulation at PC3 indicates that the visualization of PC3 with fluorescein is not attributable to a lymph node. Fluorescein, on the contrary, is a small, hydrophilic molecular dye that may follow a separate trajectory within the interstitium. The ultrasound images reveal that this pathway aligns closely with the intermuscular fascia, and based on a recent study where fluorescein was injected in low-impedance points in rats and mini-pigs [36], the migratory trajectory is located within a yet undescribed interstitial channel. Past research into lymphatics identified a separate “tissue-fluid channel” that coursed through the interstitium and represented a path of low hydraulic resistance for extracellular fluid [39, 40]. Conceivably, the fluorescein migratory pathway follows this fluid-channel route and has some functional relationship to acupuncture meridians.

The last notable observation in our study is the specificity of the migratory pathway to acupuncture points. Injection of either dye at nonacupuncture points produced only local isotropic diffusion without any associated migration into a connected linear path. Moreover, the points were injected superficially into the intradermal layer without penetration into subcutaneous depths. Somehow, the dye injected superficially at PC acupoints was able to access a deeper migratory path that enabled migration of the dye proximally along the pericardium channel. Furthermore, the dye re-emerged superficially at PC3, near the elbow, based on the strong fluorescent signal from fluorescein, which is known to be significantly attenuated at greater depths. These results reveal the possibility of a contiguous network in the forearm consisting of a deeper channel connected to superficial access points—much like what is being proposed in the meridian theory of TCM.

Collectively, the results of this experiment may help integrate the various anatomical correlates being proposed for the objective basis of acupuncture structures. The linear path undertaken by fluorescein dye aligned closely with the intermuscular fascia in the forearm, consistent with the connective tissue framework proposed by Langevin [7]. Only injection of dye at low-electrical impedance points formed these linear trajectories as revealed not only by the results of this study but also from past animal studies as well [28]. This specific, functional distinction associated with low-electrical impedance points comports with past hypotheses regarding the electrical characteristics of acupuncture points [41]. Finally, the coalescence of dye at these unique points—Evan's blue for the hypersensitized point hypothesis [16] and fluorescein dye in our study—suggests the presence of a localized pooling site within the skin that may have physiological implications. Future studies may explore whether these points have any neurovascular and interstitial convergence.

This study has several limitations. The study was limited to the pericardium channel and to healthy individuals. The generalization of this observed phenomenon may not be replicated at other body sites or other clinical scenarios. Second, the full characterization of the migration patterns—such as velocity, depth, and associated factors (e.g., hydration, arm elevation, blood pressure)—was not collected in this study. Furthermore, a rich hypergeometric statistical analysis that objectively reveals spatial concordance between tracer migration paths and acupuncturist-determined meridian/points would have substantially enhanced our ability to quantify the correlations between the two trajectories along their full lengths. Finally, the physiological significance of this dye pathway and the associated low-electrical impedance points was not explored in this study. Much remains unknown about the physiological or clinical implications of this discovered network. Future studies may explore in more detail the physiological structures located at the acupuncture points and meridians (as delineated by the fluorescein dye) and how they relate to mechanisms already elaborated by past basic science studies—such as endogenous opiate pathway. It is also necessary to quantitatively analyze the correlation between fluorescent lines and correspondent meridians regarding position and electrical impedance. Nevertheless, this study is the first to reveal a dynamic, functional link between acupuncture points and meridians in healthy humans and may open multiple avenues of research exploring the integration of the various anatomical/physiological correlates already proposed in the acupuncture sciences.

## 5. Conclusions

After injection of fluorescent dyes (fluorescein sodium and ICG) in the acupoints of the pericardium, not at adjacent nonacupoint control points, we observed their fluorescent lines proximately along this meridian. Especially fluorescein sodium emerged and ostensibly coalesced exactly where PC3 was demarcated beforehand. These lines cannot be attributed to blood vessels or lymphatics. The findings reinforce the previous studies that identified migrating pathways of tracer dyes along a linear path resembling meridians. This study may also be helpful in integrating the various anatomical correlates being proposed for the objective basis of acupuncture structures.

## Figures and Tables

**Table 1:** Comparison among injections at different points.

Injection points	Cases			*P* value, compared with nonacupoint
	Show linear path	No linear path	Total	
PC6	18	5	23	<0.001
PC5	1	0	1	0.125
PC7	3	1	4	0.024
PC5, PC6 or PC7	22	6	28	<0.001
Nonacupoint	0	7	7	—

## Data Availability

Data used to support the findings of this study are available on request from the corresponding author (Dr. Bruce Qing, tangqing@enn.cn).
